# The impact of anatomical subgroups for regional and global function of the right ventricle in hypoplastic left heart syndrome

**DOI:** 10.1186/1532-429X-13-S1-P217

**Published:** 2011-02-02

**Authors:** Carsten Rickers, Chris Hart, Michael Jerosch-Herold, Eileen Pardun, Inga Voges, Jens Scheewe, Hans Heiner Kramer

**Affiliations:** 1 University Hospital Schleswig-Holstein, Kiel, Germany; 2 Brigham and Womens Hospital, Harvard Medical School, Boston, Germany

## Introduction

The function of the right ventricle in hypoplastic left heart syndrome (HLHS) is crucial for morbidity and mortality. The aim of this study is to examine with CMR regional and global ventricular function in HLHS in relation to anatomical subgroups using MRI.

## Methods

36 children (5.8 ± 2.4 yrs) with HLHS after completion of Fontan-circulation were investigated. 17 patients (pts) had mitral and aortic atresia (MA/AA) without a visible left ventricular cavity. In 19 pts representing the anatomical subgroups mitral stenosis/aortic atresia (MS/AA) and mitral stenosis/aortic stenosis (MS/AS) a left ventricular cavity was present. We used CMR cine-imaging (TR/TE/α = 1.1/1.6/60, FOV: 240x260) for analysis of global (EF, and cardiac index-CI) and regional ventricular function. For analysis of regional (wall thickening, wall motion) ventricular function, each myocardial slice of the RV was divided into 4 anatomic segments to quantify wall thickening (%) and wall motion (mm).

## Results

In pts with MS/AA and MS/AS, wall thickening was restricted in the septal segment compared to the free wall (23.7±23.1% vs. 60.0±37.4%, p<0.001). There was no restriction in wall thickening (76.5±44.8% vs. 92.1±63.3, p=0.13) in pts without a rudimentary left ventricle (MA/AA). Analysis of regional wall motion showed that patients with MS/AA and MS/AS had also a limited wall motion in the septal segment (in relation to the other segments) as compared to pts with MA/AA (81.4±21.5% vs. 93.5±12.3%, p=0.003). The global RV-function was impaired in HLHS subgroups with a rudimentary LV (MS/AA and MS/AS), compared to those without LV (MA/AA) (CI 2.4±0.8 l/m^2^/min vs. 3.1±0.9, p=0.02).

## Conclusion

For the anatomical HLHS subtypes MS/AA and MS/AS, a rudimentary LV impairs contraction in the septal segment, resulting in a globally reduced ventricular function. This may be of prognostic significance for the long-term outcome in HLHS patients.

